# Potential Neuroprotective Effects of Adiponectin in Alzheimer’s Disease

**DOI:** 10.3390/ijms18030592

**Published:** 2017-03-09

**Authors:** Roy Chun-Laam Ng, Koon-Ho Chan

**Affiliations:** 1 Department of Medicine, LKS Faculty of Medicine, The University of Hong Kong, Hong Kong SAR, China; royclng@hku.hk; 2 Research Center of Heart, Brain, Hormone and Healthy Aging, LKS Faculty of Medicine, The University of Hong Kong, Hong Kong SAR, China; 3 Hong Kong University Alzheimer’s Disease Research Network, LKS Faculty of Medicine, The University of Hong Kong, Hong Kong SAR, China; 4 Neuroimmunology and Neuroinflammation Research Laboratory, LKS Faculty of Medicine, The University of Hong Kong, Hong Kong SAR, China

**Keywords:** adiponectin, Alzheimer’s disease, cognitive impairments, Amyloid-β

## Abstract

The adipocyte-secreted protein adiponectin (APN) has several protective functions in the peripheral tissues including insulin sensitizing, anti-inflammatory and anti-oxidative effects that may benefit neurodegenerative diseases such as Alzheimer’s disease (AD). In addition, dysregulation of cerebral insulin sensitivities and signaling activities have been implicated in AD. Emerging insights into the mechanistic roles of adiponectin and AD highlight the potential therapeutic effects for AD through insulin signaling.

## 1. Introduction

The central nervous system (CNS) is separated from the peripheral circulations by the blood-brain barrier (BBB), which stringently controls substance penetration in order to protect the brain from pathogenic micro-organisms and harmful toxins. The CNS receives and integrates information to coordinate the whole body’s activities. Intriguingly, peripheral tissues can also produce chemokines and cytokines to regulate homeostasis and metabolism in the CNS. White adipose tissue is an endocrine tissue that secretes adipokines, which exert pleiotropic effects in different tissues. Several adipokine receptors including adiponectin, leptin, and resistin receptors are abundantly expressed in different brain regions. Dysregulation of adipokine production and/or levels is associated with neurological and neurodegenerative diseases that can notably be a result of obesity-related metabolic disorders. In this review, we will discuss the plausible mechanisms of adiponectin (APN) and its protective effects in Alzheimer’s disease.

### 1.1. Association between Alzheimer’s Disease and Insulin Signaling

Alzheimer’s disease (AD) is the most common type of age-related neurodegenerative disorder, accounting for 70% of cases of dementia. Clinical symptoms include impaired spatial learning and memory decline, particularly the loss of episodic memory. Cognitive decline is a result of progressive neuronal and synaptic loss. In 1907, Alois Alzheimer described extensive accumulation of extracellular neuritic plaques and intracellular neuronal fibrillary tangles (NFTs) as two pathological changes in the brain of a dementia patient [1]. Only three decades ago, the plaque was identified as the aggregates of Amyloid-β (Aβ) peptide and the NFTs were identified as intracellular aggregates of hyperphosphorylated Tau protein [2]. These two pathologies, together with microgliosis, astrocytosis, neuronal dystrophy and synaptic reduction, represent the hallmarks of AD. In the Aβ hypothesis, Aβ is one of the major factors of AD pathogenesis as the accumulation of Aβ can induce other AD pathologies [3,4,5]. 

The formation of Aβ is caused by abnormal sequential cleavage of amyloid-precursor protein (APP) by the β- and γ-secretases, and β-site APP-cleaving enzyme 1 (BACE1) is the β-secretase essential for Aβ generation. Mutations in APP or presenilin 1 (PS1) and presenilin 2 (PS2), the subunits of γ-secretase, manifest as early onset familial AD (FAD), in which the levels of Aβ, mainly Aβ_40_ & Aβ_42_, are drastically increased [6]. The mutations may lead to either increased cleavage of APP by β-secretase or increased levels and aggregation of Aβ [7]. These familial mutations strongly support the associations between Aβ and AD. Nonetheless, FAD only accounts for 5% of AD cases. Aging is the major risk factor. Other risk factors included sporadic mutations (e.g., APOEε4), brain trauma, Down’s syndrome, vascular destruction, or abnormal metabolic functions. These factors can impede Aβ clearance and trafficking, or enhance Aβ generation, and lead to aberrant cerebral lipid metabolism and immune responses [8,9,10,11,12]. Substantial studies are ongoing in order to elucidate how these risk factors are associated with Aβ metabolism, immune responses, or cellular metabolic changes that lead to AD pathogenesis.

AD has also been described as “Type 3 Diabetes”, since reduced insulin signaling is one of the major pathophysiological mechanisms in AD patients. Certainly, Type 2 Diabetes Mellitus (T2DM) is associated with cognitive impairments and considered as a risk factor for AD [13]. There are several proposed mechanisms that link insulin to AD: (1) high insulin level (hyperinsulinemia) in diabetic patients leads to the competition of insulin degrading enzyme (IDE) with Aβ and thereby reduces the clearance of Aβ in the brain; (2) Aβ oligomers can bind to insulin receptors and block the insulin signaling pathways; (3) Aβ oligomers binding may downregulate insulin receptors via insulin receptor internalization; and (4) in obese or T2DM subjects who are characterized by increased peripheral levels of TNFα, TNFα can cross the blood-brain barrier, which results in cerebral insulin resistance due to JNK activation. Nonetheless, these hypotheses converge in the findings that deregulated insulin signaling or insulin resistance in the brain activates Glycogen Synthase Kinase 3 (GSK3), which enhances Aβ production and Tau phosphorylation. Till now, accumulated evidence including human clinical and animal model studies suggests that AD is a degenerative metabolic disorder associated with impaired cerebral insulin signaling [14].

#### Dysregulated Insulin Signaling in Clinical and Animal Studies

In 1994, Razay G. found that AD patients had peripheral hyperinsulinemia, insulin resistance and were overweight, describing the possible association between insulin signaling and AD [15]. Since then, different reports have highlighted that AD patients may have reduced insulin signaling activities in the brain. Though AD patients were found with peripheral hyperinsulinemia, AD patients have reduced levels of insulin in the cerebrospinal fluid (CSF), indicating that insulin signaling activities may be below the normal threshold in the brain [16]. This was proven by elevated levels of phosphorylated insulin receptor substrate 1 (pIRS-1) with phosphorylation at serine 616 and serine 636 residues and reduced pAkt level in the *post mortem* brain [14,17]. Lower levels of insulin receptor (IR) and insulin receptor substrate 1/2 (IRS1/2) were also found in the AD brain [18,19]. These lines of evidence support the increase of activated GSK3β levels found in AD patients [20]. AD patients have reduced CSF insulin levels that inversely correlated with Aβ_42_ levels. Late middle-aged subjects who had peripheral insulin resistance displayed reduction in cerebral glucose uptake, increased Aβ_42_ and pTau levels in CSF [21,22]. These indicated that insulin resistance and downregulated insulin signaling activities are associated with AD pathologies in patients.

The correlation between downregulated insulin signaling and AD pathology was further established in animal studies. Insulin deficiency induced by streptozotocin (STZ) in transgenic AD mice accelerated β-amyloidosis through post-translational upregulation of BACE [22]. Peripheral administration of STZ in mice reduced cerebral pAkt and pGSK3β^Ser9^ levels, and exacerbated Tau pathology, synaptic proteins reduction and memory deficits. However, STZ-treated Tau knockout mice had reduced phenotypes, indicating that insulin deficiency and reduced insulin signaling is a cause of Tau-mediated cognitive impairments [23].

### 1.2. Enhancing Insulin Signaling as a Treatment for AD

Hence, restoring cerebral insulin levels and its signaling activities has become a therapeutic target for AD treatment. It is hypothesized that increasing CNS insulin levels can improve memory functions and reduce AD phenotypes. Intranasal administration of insulin has been proposed since insulin can be degraded by digestive enzymes and therefore cannot be orally administered. This approach has been studied and has shown promising results in both animal and AD patient models [24,25,26,27,28,29]. Unfortunately, no significant change in the CSF Aβ and Tau levels were detected in AD patients after intranasal insulin treatment. Notably, there is a theoretical concern that chronic high insulin levels with prolonged intranasal insulin treatment may induce cerebral insulin resistance. This is highly possible since excessive exposure to insulin in vivo and in vitro can induce insulin resistance models with decreased pAkt and pGSK3β^Ser9^ levels.

## 2. Adiponectin

Adiponectin is an adipocyte-derived hormone that was discovered in 1995 from 3T3-L1 adipocytes [30]. It was thought to be exclusively secreted by adipose tissue until it was found to be also expressed in hepatocytes, myocytes, osteoblasts and epithelial cells. This adipokine is secreted to the circulation and functions as an endocrine hormone to regulate glucose and lipid metabolism [31]. 

APN has a molecular weight of about 30 kDa containing 244 amino acids (247 amino acids for mouse ortholog). It consists of four domains, including a signaling peptide region, a species-specific variable with cysteine residue for oligomerization, a collagenous domain comprising 22 Gly-X-Y repeats that enhances the triple helix formation in its protein structure, and a globular domain which facilitates the binding with its receptors. The full length APN protein shares structural similarities with tumor necrosis factor-α (TNFα), complement factor-1 (C1q), collagens VIII and X. The trimeric APN is the basic form in circulation that is stabilized by hydrophobic interactions between the globular domains. Trimeric adiponectin can further undergo post-translational modification to form hexamers and high molecular weight (HMW) multimers by non-covalent disulphide bonds between the cysteine Cys^36^ residues (Cys^39^ in mouse) [32,33,34]. A globular fraction, globular adiponectin (gAPN) is also found in circulation post-translationally cleaved from the full length APN monomer [35]. All of these fractions account for about 0.01% of the total protein in human serum (0.05% in rodent). However, the trimeric and globular levels are comparatively low in plasma whereas the hexameric and HMW APN are the major forms in circulation. The physiological levels of adiponectin are generally higher in females and decrease with age in both sexes [36]. Moreover, the levels of circulating APN are also affected by various conditions including hormonal and nutritional factors, the levels of circulating cytokines, disease states, chronic inflammation and exercise [37,38]. It is well known that there is an inverse association between circulating APN levels and obesity/insulin resistance as well as T2DM [39,40]. 

### 2.1. Adiponectin Receptors and Adiponectin Signaling

Adiponectin can act on various tissues to provide insulin sensitizing, anti-inflammatory, anti-oxidative and anti-atherogenic effects. Two main receptors modulate APN signaling transduction; adiponectin receptor 1 (AdipoR1) and adiponectin receptor 2 (AdipoR2). These two receptors are differentially expressed in the liver, skeletal muscle, cardiac muscle, osteoblasts, adipose tissues, pancreas, leukocytes, endothelial cells, and the brain. Adaptor protein, phosphotyrosine interacting with PH domain and leucine zipper 1 (APPL1) binds to both AdipoRs and serves as a link between receptors and the downstream signaling molecules. The signaling molecules activated by adiponectin include AMPK, p38MAPK, ERK1/2-MAPK and PPARα. Among these, AMPK acts as the main downstream effector of adiponectin. Numerous evidence has shown that Adiponectin-AdipoR1-AMPK activation provides various beneficial metabolic and protective effects to different tissues. AMPK mediates APN signaling to enhance insulin sensitivity through mTOR-p70S6K inhibition and serine phosphorylation of IRS-1 [41] in skeletal muscle. APN also activates AMPK, which phosphorylates acetyl-CoA carboxylase (ACC), enhances fatty acid oxidation, glucose uptake and utilization in liver, muscle [42] and adipocytes [43]. Signaling via AdipoR1 increases IRS-1 expression and activates APPL1 which promotes IRS-1/2 binding to insulin receptors [44,45]. Besides improving insulin sensitivity and glucose metabolism, APN exerts anti-inflammatory activities by counteracting the actions of inflammatory cytokines. APN can suppress TNFα production through the inhibition of p38MAPK and TNFα-mediated inflammatory signaling from macrophages [46,47,48]. It also blocks the action of TNFα and IL1β in lung epithelial tissues [49]. Last but not least, APN provides protection from oxidative stress-mediated cytotoxicity by reducing the production of reactive oxidative stress through AMPK signaling [50,51,52].

### 2.2. Adiponectin in the Brain

In contrast to substantial information about the roles of APN in peripheral tissues, the effect of adiponectin in the CNS is largely unknown. APN is not expressed in the brain. However, intracerebroventricular (ICV) injection of APN stimulated the paraventricular nucleus, a region involved in energy hemostasis. ICV injection of APN decreased weight gain in mice by increasing energy expenditure [53]. It was first suggested that APN was unable to cross the blood-brain barrier and could not be detected in the CSF [54,55], until several reports showed that APN was detectable in the CSF of patients with unspecified neurological disorders and in healthy subjects after IV injection of APN [56,57,58,59]. This may be due to the sensitivity of the analytical methods, as the CSF APN level is 1000-fold lower than the plasma level [56]. The distribution of APN oligomers in the CSF differs from that of the serum. Above 80% of the CSF APN is trimeric and the remaining 20% is constituted by other low molecular weight (LMW) adiponectin isoforms (e.g., hexamers) [57]. Similar experiments have been performed in mice and have shown that APN levels in the mouse CSF is 100-fold lower than plasma levels [53,60]. AdipoR1 and AdipoR2 are also highly expressed in different brain regions, including the hypothalamus, cortex, hippocampus, pituitary glands, and area postrema [61].

The physiological functions of APN in the CNS were first identified in association with food intake and body weight control [53]. It acts on the hypothalamus and activates AdipoR1-AMPK signaling to regulate food intakes, energy expenditure, and lipid and glucose metabolism during fasting [62,63]. Central administration of APN improves hypothalamic insulin signaling activities in diabetic rats and improves glucose homeostasis [64]. APN regulates neurogenesis and proliferation of hippocampal neural stem cells [60,65]. In addition, APN deficiency reduces dendritic growth and spine density in the hippocampal dentate gyrus in which the neural progenitor cells proliferation and differentiation is suppressed [66]. Besides neuroanatomical values, APN is also important in psychological functions. APN knockout (APN-KO) mice exhibit depressive-like behavior [67]. Physical activities and environmental enrichment can increase cerebral APN levels, which exert anti-depressive effects, increase hippocampal neurogenesis, and reduce neuroinflammation in mice [60,68,69]. Loss of APN in mice also exacerbates the severity of encephalomyelitis by activating lymphocytes in the mouse model of multiple sclerosis [70]. APN has anti-inflammatory and anti-atherosclerotic effects in the periphery. It is suggested that it also exerts a protective effect against ischemic brain injury. Clinical reports revealed an association between decreased APN levels and ischemic stroke. Adiponectin gene polymorphisms are associated with the risk of ischemic stroke [71,72]. Expression of adiponectin in vascular endothelial cells was increased in mice after cerebral ischemia/reperfusion injury [73]. APN treatment in mice protected the integrity of the blood-brain barrier against cerebral ischemic injury by suppressing the expression of inflammatory cytokines, and it also improved neurobehavioral performance and focal cerebral neurogenesis [74]. In summary, APN not only controls metabolism through the hypothalamic-pituitary axis, but also exerts enormous protective effects to the CNS.

## 3. Pathophysiological Roles of Adiponectin in Alzheimer’s Disease

Adiponectin has protective effects to the CNS. Studies have been conducted to investigate the roles of adiponectin in neurodegenerative disease. In 2011, Une et al. found increased CSF APN in patient with mild cognitive impairment (MCI) but not in AD patients, when compared with control subjects [75]. A year after the report by Une et al. was published, a prospective clinical study concluded the increased plasma adiponectin level is an independent risk factor for dementia and AD in women [76]. Texixeira A.L. and his colleagues found reduced serum adiponectin levels in MCI and AD patients with similar subject size [77]. More recently, Wennberg et al. found that the serum adiponectin is positively correlated to the levels of amyloid but inversely correlated to the hippocampal volume in women with MCI [78]. Waragi et al. revealed a reduction of CSF APN levels in AD patients correlating to an increase of CSF Aβ_42_, CSF p-Tau and the presence of hippocampal atrophy. This research group also suggested that an increase of circulating adiponectin levels could be a compensatory effect against neurodegeneration [79]. In contrast to previous findings, another report showed no significant difference of adiponectin levels between a non-demented group and patients with dementia [80]. Furthermore, Yu et al. found correlations between adiponectin gene polymorphism and late onset AD [81]. Two studies reported on low adiponectin levels in T2DM patients who had reduced gray matter volume, hippocampal volume and glucose metabolism, to be consistent with Alzheimer’s disease pathophysiologies [79,82]. Another group reported that T2DM patients with MCI had lowered serum adiponectin levels [83]. Besides human patients, Kurata et al. showed the reduction of serum APN levels in APP transgenic mice [84]. Adiponectin transport to the brain decreased with age while peripheral APN levels showed no difference between young and aged rats [85]. This indicated that the cerebral APN may be important for memory and learning. However, these clinical studies cannot provide insight regarding the causality of adiponectin to AD. These studies only revealed associations or correlations, and showed variation among different study groups. No cause-and-effect study had been conducted until we recently reported that chronic adiponectin deficiency elicited AD-like phenotypes, pathologies and cognitive impairments with cerebral insulin resistance in aged mice [86].

**Figure 1:**
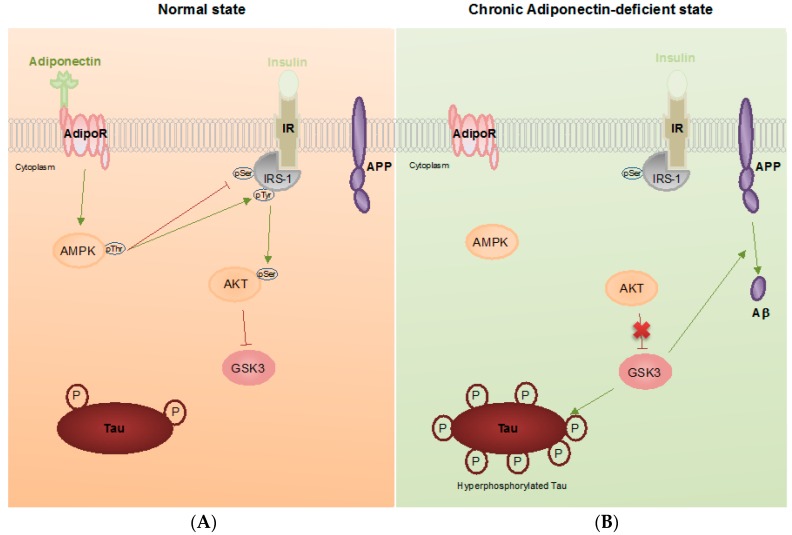
Pathophysiological roles of adiponectin in Alzheimer’s disease (AD). Schematic outline of neuronal adiponectin signaling in the normal brain (**A**); and adiponectin-deficient brain (**B**). Under physiological conditions, adiponectin binds to its receptor and triggers phosphorylation of AMPK which inhibits IRS-1 phosphorylation at serine residues. This increases insulin-mediated IRS-1 phosphorylation at tyrosine residues and promotes downstream Akt-mediated GSK3 inhibition. Inhibition of GSK3 slows down phosphorylation of Tau and APP metabolism. In AD, chronic adiponectin deficiency leads to an increase of IRS-1 phosphorylation at serine residues (e.g., Serine 616). This causes reduced pIRS-1Tyr and results in GSK3 activation. Activated GSK3 enhances Tau phosphorylation and Aβ production in neurons. Arrows denote promotion, T-bars denote inhibition.

In 2012, we showed that APN was neuroprotective against oxidative stress-induced cytotoxicity under Aβ toxicity in vitro [87]. We hypothesized that hypoadiponectinemia may lead to Aβ accumulation, which is neurotoxic and may result in neurodegeneration. Recently, we found that aged mice with chronic adiponectin deficiency had memory decline, spatial learning impairment, increased anxiety, and impaired contextual fear conditioning. Deregulated cerebral insulin signaling activities and reduced hippocampal insulin sensitivity developed during the aging of APN-KO mice. Aberrant activation of GSK3β was revealed by reduced pGSK3β^S9^ and increased pGSK3β^Y279^ levels in aged APN-KO mice. These findings provide an explanation for the dramatic increase of phosphorylated Tau, increased Aβ42 production and Thioflavin S positively stained Aβ deposition. Intriguingly, we detected Aβ*56 in the brain of aged APN-KO mice whereas it was absent in the brain of the wild-type mice. Moreover, we found increased microgliosis, astrogliosis with increased cerebral TNFα and IL1β levels that are the common hallmarks of AD. The reduced synaptic proteins levels and increased neuronal apoptosis also indicated that chronic adiponectin deficiency was associated with neurodegeneration in aging (Figure 1). Adiponectin-treated SH-SY5Y neuroblastoma cells carrying APP_swedish_ mutation had reduced extracellular Aβ42 peptide. SH-SY5Y neuroblastoma cells treated with high concentration of insulin displayed insulin resistance with reduced pAkt level. The insulin sensitivity of the insulin resistant SH-SY5Y was restored after treatment with exogenous trimeric adiponectin protein through the activation of AMPK. Our findings provide strong evidence that reduced adiponectin is associated with deregulated cerebral insulin signaling and AD pathogenesis in aged or T2DM subjects who have decreased CNS adiponectin levels [86]. 

It is suggested that rodents do not develop amyloid pathologies, probably because of the longevity and low aggregating propensity of rodent Aβ. Mice with a specific gene knocked out or carrying mutated human APP or PS transgene have increased Aβ deposition and generation. We found that the aged APN-KO mice had increased GSK3β activity with increased Aβ production. A recent report indicates that the inhibition of GSK3β activity can decrease BACE1 expression, which results in reduced Aβ production [88]. We also found increased formation of a specific Aβ oligomer (Aβ*56) in aged APN-KO mice with no dimeric or trimeric Aβ being detected. Aβ*56 is a specific Aβ oligomer found in patients with MCI and transgenic AD mice at a young age [89]. Aβ*56 leads to dose-dependent cognitive decline in mice, whereas trimeric Aβ does not [90]. Though the detailed mechanism of how adiponectin regulates the formation of Aβ*56 remains unknown, adiponectin deficiency promotes rodent Aβ production through GSK3β-mediated activities.

In summary, it is hypothesized that the decrease of APN levels or reduction in adiponectin signaling activities can be a cause of AD pathogenesis and cognitive impairments. The association is highly linked to the deregulated insulin signaling activities and reduced insulin sensitivity in the brain.

## 4. Therapeutic Potential of Adiponectin

Effective treatment or medication for AD is currently limited. Donepezil is one of the four acetylcholinesterase inhibitors that are marketed drugs for the symptomatic treatment of AD. Intriguingly, the serum adiponectin levels progressively increased in AD patients who were administered with Donepezil [91]. Adiponectin has been considered as a therapeutic target to treat metabolic syndromes because of its potent protective effects to both the CNS and peripheral tissues. However, adiponectin cannot be orally administered due to its protein nature. Intravenous injection of recombinant adiponectin protein can by-pass the digestive system, but prolonged injection treatments are costly. The inventions of pharmacological agents or changes of lifestyle to elevate endogenous adiponectin expression or activate adiponectin signaling may pave the road for future AD treatment.

**Figure 2:**
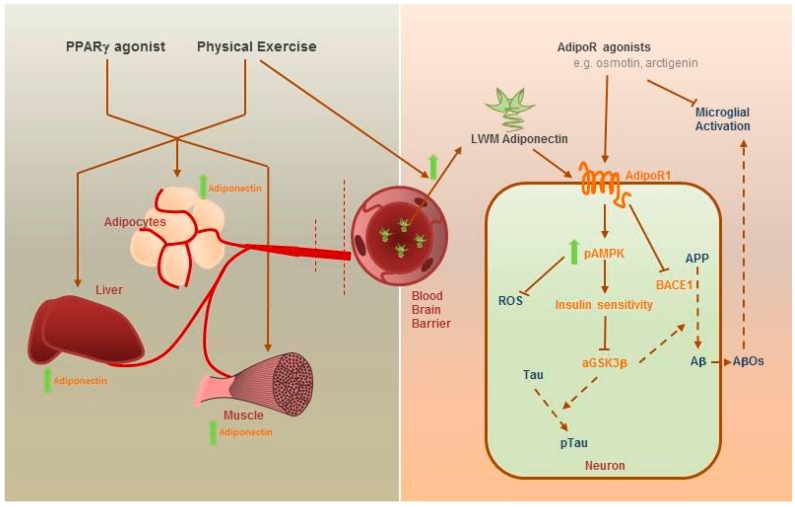
Strategies to increase adiponectin signaling in AD neurons. Peripherally, adiponectin expression from the liver, adipocytes and muscle can be increased by administering PPARγ agonist (e.g., pioglitazone) and physical exercise induction. Physical exercise can also increase the transport of low molecular weight nd trimeric adiponectin across the blood-brain barrier (BBB). Administration of adipoR agonists and LMW adiponectin acts on AdipoR1 in neurons and microglia to exert neuroprotection. Green arrow denotes increase, arrows and dash arrows denote promotion, T-bars denote inhibition.

Physical exercise has long been considered to exert beneficial effects on brain functions, improve psychiatric symptoms and delay progression of neurodegenerative diseases. Increase of peripheral adiponectin levels and transport of adiponectin across the BBB were reported in rodents and human with increased physical exercise. Peroxisome proliferator-activated receptor γ (PPARγ) agonist increases adiponectin expression and exerts insulin-sensitizing effects. PPARγ agonist such as pioglitazone is an approved medication to T2DM patients. Repositioning of pioglitazone to treat AD is under investigation. AD mice fed with pioglitazone have shown cognitive improvements and suppressed microglial reactivation. However, it is known that pioglitazone crosses the BBB weakly and whether it works through adiponectin to exert neuroprotection remains uncertain (Figure 2).

More recently, several adiponectin receptor agonists have been discovered. In 2013, several adiponectin receptor 1 agonists were discovered including matairesinol, arctiin, arctigenin and gramine from the screening of 10,000 natural compounds [92]. Concomitantly, in the same year, arctigenin was found to improve memory functions in the AD mice model that suppressed Aβ production by inhibiting BACE1 activity and enhanced autophagic clearance of Aβ by Akt-mTOR inhibition and AMPK activation. Last but not the least, osmotin, a plant homolog of mammalian adiponectin, was identified as a novel adipoR1 agonist which is neuroprotective against ethanol-induced apoptosis and glutamate-induced synaptic dysfunction in rat brain. It also suppresses Aβ-induced memory impairments, Tau phosphorylation and neurodegeneration in mouse hippocampus. Furthermore, it modulates microglia activation against LPS-induced neuroinflammation through AdipoR1-TLR4/NF-κB signaling cascade [93]. Notably, osmotin was found to improve synaptic functions and cognitive functions, and diminish Aβ production and aggregation through AdipoR1-AMPK-SIRT1 pathway.

## 5. Conclusions 

It is indicated that hypoadiponectinemia in the brain may be implicated in neurodegeneration during aging. The reduced adiponectin levels in T2DM patients may be a factor which increases the risk of AD. T2DM patients or subjects with chronic adiponectin deficiency may develop cerebral insulin resistance. Decreased cerebral insulin signaling activities promotes Aβ production and Tau phosphorylation, causing neuroinflammation and neurodegeneration. Regarding its neuroprotective roles including insulin sensitizing, anti-inflammatory and anti-oxidative effects, adiponectin may pave the road to prevent and treat Alzheimer’s disease. 

## Abbreviations

AD	Alzheimer’s disease
Aβ	Amyloid-β
AdipoR	Adiponectin receptor
AMPK	5′ adenosine monophosphate-activated protein kinase
APN	Adiponectin
APP	Amyloid precursor protein
APPL1	Adaptor protein, phosphotyrosine interacting with PH domain and leucine zipper 1
BACE1	β-site APP cleaving enzyme 1
BBB	Blood-brain barrier
CNS	Central nervous system
ERK1/2	Extracellular signal-regulated kinase1/2
FAD	Familial Alzheimer’s disease
GSK	Glycogen synthase kinase
HMW	High molecular weight
ICV	Intracerebroventricular
IV	Intravenous
IDE	Insulin degrading enzyme
IL1β	Interleukin 1β
IR	Insulin receptor
IRS-1	Insulin receptor substrate 1
JNK	c-Jun N-terminal kinase
LMW	Low molecular weight
LPS	Lipopolysaccharide
MAPK	Mitogen-activated protein kinases
MCI	Mild cognitive impairment
NFT	Neurofibrillary tangles
mTOR	mammalian target of rapamycin
NF-κB	Nuclear factor κ B
PPARα	Peroxisome proliferator-activated receptor α
PPARγ	Peroxisome proliferator-activated receptor γ
PS	Presenilin
SIRT-1	NAD-dependent deacetylase sirtuin-1
T2DM	Type 2 diabetes mellitus
TLR4	Toll-like receptor 4
TNFα	Tumor necrosis factor α
